# Sequencing a Strawberry Germplasm Collection Reveals New Viral Genetic Diversity and the Basis for New RT-qPCR Assays

**DOI:** 10.3390/v13081442

**Published:** 2021-07-24

**Authors:** Alfredo Diaz-Lara, Kristian A. Stevens, Vicki Klaassen, Min Sook Hwang, Maher Al Rwahnih

**Affiliations:** 1 Department of Plant Pathology, University of California-Davis, Davis, CA 95616, USA; diazlara@tec.mx; 2 School of Engineering and Sciences, Tecnologico de Monterrey, Campus Queretaro, Queretaro 76130, Mexico; 3 Foundation Plant Services, University of California-Davis, Davis, CA 95616, USA; kastevens@ucdavis.edu (K.A.S.); vaklaassen@ucdavis.edu (V.K.); mshwang@ucdavis.edu (M.S.H.); 4 Department of Computer Science, University of California-Davis, Davis, CA 95616, USA; 5 Department of Evolution and Ecology, University of California-Davis, Davis, CA 95616, USA

**Keywords:** strawberry, germplasm, virome, high throughput sequencing, virus, detection, RT-qPCR, diagnostic assay

## Abstract

Viruses are considered of major importance in strawberry (*Fragaria* × *ananassa* Duchesne) production given their negative impact on plant vigor and growth. Strawberry accessions from the National Clonal Germplasm Repository were screened for viruses using high throughput sequencing (HTS). Analyses of sequence information from 45 plants identified multiple variants of 14 known viruses, comprising strawberry mottle virus (SMoV), beet pseudo yellows virus (BPYV), strawberry pallidosis-associated virus (SPaV), tomato ringspot virus (ToRSV), strawberry mild yellow edge virus (SMYEV), strawberry vein banding virus (SVBV), strawberry crinkle virus (SCV), strawberry polerovirus 1 (SPV-1), apple mosaic virus (ApMV), strawberry chlorotic fleck virus (SCFaV), strawberry crinivirus 4 (SCrV-4), strawberry crinivirus 3 (SCrV-3), Fragaria chiloensis latent virus (FClLV) and Fragaria chiloensis cryptic virus (FCCV). Genetic diversity of sequenced virus isolates was investigated via sequence homology analysis, and partial-genome sequences were deposited into GenBank. To confirm the HTS results and expand the detection of strawberry viruses, new reverse transcription quantitative PCR (RT-qPCR) assays were designed for the above-listed viruses. Further in silico and in vitro validation of the new diagnostic assays indicated high efficiency and reliability. Thus, the occurrence of different viruses, including divergent variants, among the strawberries was verified. This is the first viral metagenomic survey in strawberry, additionally, this study describes the design and validation of multiple RT-qPCR assays for strawberry viruses, which represent important detection tools for clean plant programs.

## 1. Introduction

The garden or commercial strawberry (*Fragaria* × *ananassa* Duchesne) is a widely grown hybrid species of the genus *Fragaria* (family Rosaceae), which is cultivated as a source of food in many parts of the world [1]. This plant was first bred in Europe around 1750 and is a hybrid between *F. virginiana* from North America and *F. chiloensis* from South America [2,3]. The United States is one of the major worldwide producers of strawberry with 1.2 million tons in 2018, produced primarily in the state of California (Food and Agriculture Organization. http://www.fao.org/faostat/en/#data/QC, accessed on 26 October 2020). California strawberries are available year-round because of the mild climate, which allows harvesting fruits over a long period of time [4].

An important resource of genetic material from *Fragaria* is the US Department of Agriculture, ARS National Clonal Germplasm Repository (NCGR) located in Corvallis (OR, USA). The NCGR contains 2013 active strawberry accessions, representing 46 taxa including species and subspecies (GRIN-Global. https://npgsweb.ars-grin.gov/gringlobal/search, accessed on 27 October 2020). Besides having foundational material for horticultural distribution, the NCGR also separately houses pathogen-positive accessions for virology research.

Various viral diseases have been reported in strawberry and are associated with plant decline and yield loss (for a review [5,6]). The high number of identified viruses in strawberry is the result of vegetative propagation and exposure of the plant in open-field cultivation. Additionally, different vectors (e.g., insects and nematodes) have been shown to transmit these viruses. Lastly, viruses in strawberry plants may be in low concentrations and in mixed infections, and commonly induce non-specific symptoms [7].

Reliable and efficient diagnostic tests are critical in determining viral infection in strawberry and consequently the control of disease. Multiple laboratory-based tests are available for the diagnosis of viruses in strawberry, mainly involving ELISA and reverse transcription PCR (RT-PCR), previously reviewed in [8]. In contrast, the use of quantitative RT-PCR (RT-qPCR) assays for strawberry viruses is extremely limited or absent. The advantages of using RT-qPCR over other classical molecular-serological methods to detect viruses are increased sensitivity, speed, reproducibility, and limited risk of contamination [9].

Recently, next generation sequencing or high throughput sequencing (HTS) has been used to reveal the etiology of several diseases in strawberry. For example, a novel virus (i.e., strawberry polerovirus-1, SPV-1) was associated with the strawberry decline disease in Canada [10]. Two new putative viruses in the genus *Crinivirus*, strawberry criniviruses 3 and 4 (SCrV-3 and 4), were identified in strawberry plants displaying virus-like symptoms [11,12]. In 2019, Fránová et al. [7] sequenced a novel rhabdovirus infecting garden and wild strawberry plants.

To further investigate the virome of strawberry, we analyzed 45 plants collected at the NCGR by HTS. Multiple variants of viruses known to infect strawberry were annotated and characterized, including near-complete genomes. To complement these HTS results, new RT-qPCR assays were developed and validated for 14 different viruses.

## 2. Materials and Methods

### 2.1. Plant Material and TNA Extraction

**Figure 1:**
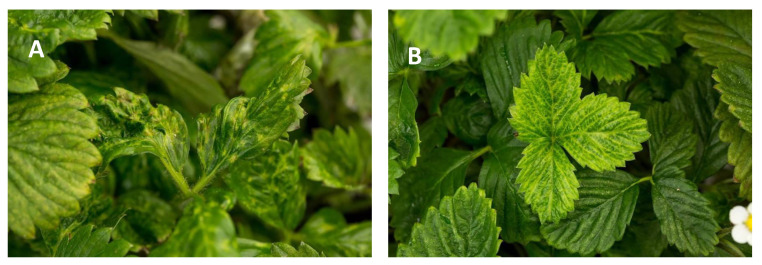
Virus-like symptoms on select strawberry plants that were sampled during the study. (**A**), plant infected by beet pseudo yellows virus and strawberry crinkle virus; (**B**), plant infected by strawberry vein banding virus.

In the summer of 2020, 45 accessions of strawberry were received as propagative material from the NCGR, under a USDA Animal and Plant Health Inspection Service (APHIS) movement permit, for HTS analysis at Foundation Plant Services (FPS, University of California-Davis, Davis, CA, USA). These 45 plants were previously determined to be positive for viruses or virus-like agents using RT-PCR and bio-indexing. Stolons were propagated under mist and later transferred to single 1-gal pots within a greenhouse. Four months after bud break, 0.7 g of leaf tissue from each strawberry plant was collected and spiked with 5% (*w/w*) tissue of *Phaseolus vulgaris* cultivar Black Turtle Soup (BTS). BTS is naturally infected by two different endornaviruses and is used as an internal control in HTS virus screening [13]. At the time of sampling different leaf symptoms were observed in several strawberry plants (Figure 1). Following the protocol described in [14], total nucleic acid (TNA) extracts were prepared using guanidine isothiocyanate lysis buffer and a KingFisher Flex System with the MagMax™ Plant RNA Isolation kit (ThermoFisher Scientific, Sunnyvale, CA, USA).

### 2.2. HTS and Plant Virus Identification

For individual samples, a total of 700 ng per 10 µL of extracted nucleic acids were subjected to rRNA and cDNA library construction using TruSeq Stranded Total RNA with Ribo-Zero Plant kit (Illumina, San Diego, CA, USA). Subsequently, cDNA libraries were end-repaired, adapter-ligated by unique dual-indexes, and PCR enriched. Finally, the amplicons were sequenced in an Illumina NextSeq 500 platform using a single-end 75-bp format.

Sequenced reads were demultiplexed and adapter trimmed using Illumina bcl2fastq2 v2.20.0.422. To obtain the highest quality contig for an annotated virus, three de novo assemblies were performed: (1) a de novo assembly using SPAdes v3.14.1 [15]; (2) a de novo assembly using SPAdes v3.14.1 in RNA; and (3) an overlap layout consensus assembly of the virus annotated contigs from (1) using the Minimo assembler [16]. For each virus infection, the assembly yielding the longest contigs was chosen.

To annotate de novo assemblies, all contigs greater than 200 bp were aligned to the June 2020 version of the GenBank non-redundant database of nucleotide sequences using BLASTn with a reduced word size of 7 to identify known viruses. Additionally, to screen for potential unknown viruses, contigs greater than 200 bp were aligned to the June 2020 version of the GenBank non-redundant database of nucleotide sequences using BLASTx. Then, sequences with significant hits (*E*-value < 1 × 10^−5^) in this database to a virus known to infect land plants went through a final manual annotation check.

### 2.3. Plant Virus Genome Analysis

Potential open reading frames (ORFs) and proteins encoded by the HTS-detected viruses were annotated by ORFfinder and BLASTp analysis. Conserved domains present in the putative proteins were searched in the Pfam database [17] using HMMER v3.1 [18]. Once sequence analysis was completed, new virus sequences were deposited in GenBank.

### 2.4. RT-qPCR Assay Design

Following the protocol described in [19], new RT-qPCR assays were designed for all the viruses identified during the HTS analysis. Using a comprehensive approach that covers all the known virus genetic diversity in this study and in GenBank, detection assays based on sequence-specific DNA hydrolysis probes (TaqMan™ MGB) were generated. Determination of primer and probe sequences for a target region included the default parameters for qPCR in the Primer Express software (ThermoFisher Scientific, Sunnyvale, CA, USA).

After initial assay design was completed, in silico analysis facilitated by purpose-built scripts implementing the procedures described below was used to incorporate additional viral genetic diversity into assay design. For each of the viruses, the RT-qPCR assay was first evaluated against all virus sequences in the July 2020 version of GenBank as well as all virus isolates identified during this study. First, a BLAST database search is used to identify and obtain all sequences overlapping the current assay region. To maximize sensitivity, a tBLASTn translated alignment exploiting codon redundancy was used. Once target sequences were collected and their species identification confirmed, all existing primers and probes were aligned to all target sequences covering the assay region. This alignment was accomplished using a script that used an end-gap-free nucleotide alignment to identify the best matching probe, forward and reverse primer sequences to each variant. In each case, the variant sequences corresponding to the matching oligos were collected and analyzed for divergence. Thus, all unique candidate sequence variants were inspected for total or partial divergence to an existing primer/probe sequence. The location and quantity of nucleotide differences and the frequency of the differences were also determined, and the assays were updated with extra primers or probes as needed. A probe or primer was added when more than two nucleotide mismatches or a single mismatch near the end were detected during the sequence comparison. Lastly, in order to reduce the effective number of primers in each reaction, degenerate bases were not used in the oligos.

Once assay design was completed, their efficiency was calculated via serial dilutions of 1:1 to 1:10,000,000 and replicated in triplicate; standard curves were generated by the QuantStudio 6 Flex Real-Time PCR System software (ThermoFisher Scientific, Sunnyvale, CA, USA).

### 2.5. HTS Validation by RT-qPCR

Leaf tissue was collected from all the propagated strawberry plants and TNA was extracted as described above but reducing the input material to 0.2 g and omitting the addition of BTS. RT-qPCR reactions were completed in the QuantStudio 6 Flex Real-Time PCR System using the TaqMan Fast Virus 1-Step Master Mix (ThermoFisher Scientific, Sunnyvale, CA, USA) as per manufacturer’s protocol. Each reaction (10 µL final volume) included 2 µL of TNA and final primer and probe concentrations of 900 and 250 ῃM, respectively. In addition, the new assays were multiplexed with an 18S rRNA assay to confirm the presence of RNA [20].

### 2.6. Additional Testing by RT-qPCR

The new RT-qPCR assays were used to test 12 virus-positive strawberry plants located at FPS. Given the nature of FPS as a center for distribution of virus-tested propagation material, occasionally plants infected by viruses are identified and adopted as positive controls in routine screening (https://fps.ucdavis.edu/index.cfm, accessed on 1 July 2021). All 12 plants were previously analyzed by RT-PCR and HTS, revealing the presence of several viruses included in this study.

## 3. Results

### 3.1. Viral Sequences Identified by HTS

Forty-five strawberry plants originating from the NCGR were screened for viruses via HTS. HTS yielded consistent results for the sequencing, assembly, and annotation of each of the samples (Table S1). Across multiple runs, the Illumina sequenced read depth for each ranged from 23.4 to 32.2 million 75-bp reads with a median value of 28.0 million and a coefficient of variation of 0.09. More variation was observed in the subsequent Spades de novo assemblies of the metagenomes, which ranged from 9.0 Mbp to 30.0 Mbp contigs with a median value of 20.5 Mbp and a coefficient of variation of 0.27. The number of contigs that annotated as viral was a small fraction (0.01–0.25%) of each assembly.

Ignoring the multiple endornavirus-like contigs generated in all the HTS-analyzed samples, no plant infecting viruses were identified in five plants but viruses in single and mixed infections were identified in the remaining 40 plants (Table 1). These viruses included: strawberry mottle virus (SMoV), beet pseudo yellows virus (BPYV), strawberry pallidosis-associated virus (SPaV), tomato ringspot virus (ToRSV), strawberry mild yellow edge virus (SMYEV), strawberry vein banding virus (SVBV), strawberry crinkle virus (SCV), apple mosaic virus (ApMV), strawberry chlorotic fleck virus (SCFaV), Fragaria chiloensis latent virus (FClLV), Fragaria chiloensis cryptic virus (FCCV), SCrV-3, SCrV-4, and SPV-1.

A separate annotation was performed to identify potential novel plant viruses in these samples, characterized by divergent protein homology to a virus known to infect plants. No such sequences were observed. However, distant protein homology to insect viruses, mycoviruses and phages was observed.

### 3.2. New Viral Genetic Diversity

The BLASTn annotation of the assembled contigs revealed a genetically diverse set of sequences from plant viruses known to infect strawberry. We identified the genomes of 14 plant viruses, represented by 65 different isolates (ApMV, 3; BPYV, 14; FCCV, 3; FClLV, 4; SCFaV, 1; SCrV-3, 3; SCrV-4, 3; SCV, 2; SMoV, 4; SMYEV, 14; SPaV, 5; SPV-1, 5; SVBV, 3; ToRSV, 1) among the 45 samples we analyzed. For each distinct infection (i.e., plant virus per sample), we selected the longest sequence as representative of the infecting isolate. Thus, a total of 65 partial sequences, including several near complete genomes, were annotated for their protein coding regions and deposited in GenBank.

We used the nucleotide identity to the closest homolog in GenBank as an estimate of the amount of additional nucleotide diversity each of these sequences provide. As indicated in Table 2, this study provides substantial new diversity for six of the 14 strawberry infecting viruses. Further, if we consider 90% nucleotide identity as the cutoff for a divergent isolate, we obtained a total of 19 new divergent isolates (BPYV, 1; SCrV-4, 2; SMoV, 1; SMYEV, 10; SPaV, 4; ToRSV, 1). The virus with the greatest amount of nucleotide diversity and largest number of divergent isolates was SMYEV, a positive sense RNA virus. In contrast, the virus with the lowest amount of additional nucleotide diversity, based on three sequenced isolates, was SVBV, a DNA genome virus.

### 3.3. New RT-qPCR Assays

New RT-qPCR assays were developed for SMoV, BPYV, SPaV, ToRSV, SMYEV, SVBV, SCV, SPV-1, ApMV, SCFaV, SCrV-4, SCrV-3, FClLV and FCCV. According to the in silico analysis, most assays needed multiple forward and reverse primers and/or probes (Table 3) to cover all the known genetic diversity of these viruses. To reduce the number of potential primer combinations, we preferred using multiple primers to degenerate bases. The amplification efficiency varied among assays and ranged from 87.5% to 118.6% (Figure S1).

### 3.4. Detection of Viruses by RT-qPCR

To confirm the presence of viruses identified by HTS, source plants were analyzed by newly designed RT-qPCR assays. Virus detection was then validated by comparing HTS and RT-qPCR results for each virus-infected sample. In all cases, RT-qPCR and HTS results agreed (Table S2). Likewise, no amplification was observed in samples H2390, H2397, H2411, H2412 and H2430, confirming the virus-free status of these plants.

To further validate the new assays, twelve FPS positive control plants were analyzed by both HTS and RT-qPCR (Table S3). The following viruses were identified by both methods: SMoV (four plants), BPYV (seven plants), SPaV (five plants), SMYEV (three plants), SVBV (two plants), and SPV-1 (three plants).

## 4. Discussion

The primary objectives of this study were to increase available genetic diversity of strawberry viruses by characterizing a diverse set of infected plants and to develop a comprehensive set of RT-qPCR assays for detecting viruses infecting strawberries. The new sequence resources were utilized in the design of the detection assays.

The NCGR provided us with a vast collection of accessions of geographically and genetically diverse provenance. From that collection, all strawberry samples identified as pathogen-infected were included in a viral metagenomic survey using HTS. This approach allowed us to efficiently obtain sequences for all viruses infecting the sample. It is the first such survey of this type in strawberry, templated on work done in previous crops [21,22,23]. The metagenomic analysis revealed the presence of 14 different strawberry infecting viruses, the majority of which had RNA genomes. More detailed HTS analyses indicated that 40 out of 45 plants were infected with at least one virus and 34 had mixed infections of up to five different viruses.

Considerable additional genetic diversity was observed in several cases of strawberry viruses. In total, 65 partial genome sequences from 14 strawberry-infecting viruses were deposited in GenBank. These represent a substantial contribution to sequence resources for strawberry viruses, increasing the number of sequences deposited in GenBank by approximately 10%. Moreover, these sequences extend the genetic diversity of strawberry infecting viruses characterized in previous amplicon-based studies [24,25,26,27]. Notably, our most highly divergent population of new virus isolates comes from SMYEV. Xiang et al. [28] also observed multiple highly divergent populations of SMYEV in their amplicon-based study. The lowest amount of additional genetic diversity was observed in SVBV; this result is also consistent with the lower mutation rates observed for DNA viruses [29].

Four different criniviruses infecting strawberry were found during the metagenomics survey (BPYV, SPaV, SCrV-3 and -4). Most of these viruses displayed considerable genetic diversity, with the exception of SCrV-3, which lacked divergent variants. In that sense, an outstanding question we believe needs to be addressed is whether SCrV-3 and SCrV-4 represent new virus species or whether they are strains of a known virus. Novel data, like those generated here, will help to understand the biology and molecular biology of criniviruses, which remains to be fully understood [30].

Utilizing these new genetic resources, a comprehensive set of new RT-qPCR detection assays were designed for strawberry viruses. New assays were validated in vitro to confirm their reliability. For example, twelve strawberry samples previously screened for viruses and located at FPS were retested using the newly designed RT-qPCR assays. While we did not compare the sensitivity of our new assays with other PCR-based methods such as end-point RT-PCR, it is generally accepted that RT-qPCR is a highly sensitive method. Multiple primers and probes were employed to detect diverse virus isolates, as demonstrated with a similar assay for grapevine leafroll-associated virus 3, a highly divergent virus infecting grapevine [31].

The high throughput nature of RT-qPCR is desirable for clean plant certification facilities that process large sample numbers. Except for previous work describing RT-qPCR assays for strawberry necrotic shock virus and SCV, there is a scarcity of published assays of this kind for detecting strawberry viruses [32,33]. The novel diagnostic tools described here address that scarcity, looking to improve the management of strawberry viruses in the United States and globally.

## Figures and Tables

**Table 1:** Viruses identified in strawberry plants via high throughput sequencing (HTS). Plants originated from the National Clonal Germplasm Repository (NCGR) located in Corvallis, Oregon.

Sample ID ^a^	SMoV	BPYV	SPaV	ToRSV	SMYEV	SVBV	SCV	SCFaV	SCrV-4	SPV-1	ApMV	SCrV-3	FClLV	FCCV
H2389	+													
H2390														
H2391		+	+	+										
H2392				+	+									
H2393						+								
H2394		+			+									
H2395		+					+							
H2396	+	+												
H2397														
H2398					+									
H2399		+	+				+	+	+					
H2400					+					+				
H2401		+									+			
H2402		+	+		+				+					
H2403	+	+									+			
H2404	+	+								+				
H2405	+	+												
H2406			+		+				+			+		
H2407		+	+											
H2408		+			+									
H2409		+								+				
H2411														
H2412														
H2413		+									+			
H2414		+												
H2415		+					+							
H2416		+					+							
H2417		+					+							
H2418			+						+					
H2419		+	+											
H2421			+			+								
H2422					+									
H2423					+									
H2427													+	+
H2424	+				+		+			+				
H2425					+								+	+
H2426					+								+	
H2428					+					+			+	+
H2430														
H2429					+					+				
H2431						+								
H2432			+		+				+			+		
H2433		+												
H2434					+								+	
H2435			+		+				+			+		

Strawberry mottle virus (SMoV), beet pseudo yellows virus (BPYV), strawberry pallidosis-associated virus (SPaV), tomato ringspot virus (ToRSV), strawberry mild yellow edge virus (SMYEV), strawberry vein banding virus (SVBV), strawberry crinkle virus (SCV), strawberry polerovirus 1 (SPV-1), apple mosaic virus (ApMV), strawberry chlorotic fleck virus (SCFaV), strawberry crinivirus 4 (SCrV-4), strawberry crinivirus 3 (SCrV-3), Fragaria chiloensis latent virus (FClLV) and Fragaria chiloensis cryptic virus (FCCV); ^a^, sample IDs assigned to different strawberry plants analyzed by HTS.

**Table 2:** New isolate sequences of strawberry-infecting viruses and their closest homolog in GenBank.

Virus	Isolate	ID	Top Hit ID	%ID	% Cov	Virus	Isolate	ID	Top Hit ID	%ID	% Cov
ApMV	H2401	MZ320192	KY965059.1	92	100	SMoV	H2389	MZ326670	KU200461.1	83	99
	H2403	MZ320193	KY965059.1	92	99		H2396	MZ326671	MH746440.1	97	99
	H2413	MZ320194	KY965059.1	92	100		H2404	MZ326672	LC550286.1	94	99
BPYV	H2416	MZ320195	AY330918.2	83	98		H2405	MZ326673	LC550287.1	96	100
	H2433	MZ320196	AY330918.2	100	100	SMYEV	H2423	MZ326674	D12517.1	99	100
	H2391	MZ320197	AY330918.2	100	100		H2424	MZ326675	KR559736.1	80	100
	H2396	MZ320198	AY330918.2	99	100		H2425	MZ326676	LC515236.1	82	99
	H2401	MZ320199	AY330918.2	99	100		H2429	MZ326677	KR559736.1	79	99
	H2402	MZ320200	AY330918.2	99	100		H2432	MZ326678	KR350470.1	89	100
	H2403	MZ320201	AY330918.2	99	100		H2435	MZ326679	KX150372.1	82	100
	H2405	MZ320202	AY330918.2	100	100		H2470	MZ326680	KR559736.1	79	99
	H2407	MZ320203	AY330918.2	100	100		H2394	MZ326681	KR559736.1	80	100
	H2408	MZ320204	AY330918.2	99	100		H2398	MZ326682	LC515236.1	97	100
	H2409	MZ320205	AY330918.2	99	100		H2400	MZ326683	D12517.1	91	100
	H2413	MZ320206	AY330918.2	100	100		H2402	MZ326684	KR559736.1	80	100
	H2415	MZ320207	AY330918.2	100	100		H2406	MZ326685	KR350470.1	88	100
	H2419	MZ320208	AY330918.2	100	100		H2408	MZ326686	AJ577359.1	98	100
FCCV	H2425	MZ320209	DQ093961.2	100	100		H2422	MZ326687	KR350470.1	85	100
	H2427	MZ320210	DQ093961.2	100	100	SPaV	H2435	MZ351164	AY488138.2	100	99
	H2428	MZ320211	DQ093961.2	99	99		H2391	MZ351165	MN747001.1	86	63
FCILV	H2425	MZ320212	AY682102.1	100	100		H2407	MZ351166	AY488137.2	79	71
	H2426	MZ320213	AY682102.1	100	99		H2419	MZ351167	MN747001.1	88	99
	H2427	MZ320214	AY682102.1	95	100		H2420	MZ351168	MN747001.1	90	97
	H2428	MZ320215	AY682102.1	95	100	SPV-1	H2429	MZ351169	KM233706.1	97	100
SCFaV	H2399	MZ320216	DQ860839.1	100	100		H2470	MZ351170	MK142237.1	98	99
SCrV-3	H2432	MZ326662	EU267168.1	100	91		H2400	MZ351171	KM233706.1	97	100
	H2435	MZ326663	EU267168.1	100	92		H2404	MZ351172	KM233705.1	99	100
	H2406	MZ326664	EU267168.1	96	91		H2409	MZ351173	KM233705.1	98	100
SCrV-4	H2432	MZ326665	KY488557.1	83	77	SVBV	H2431	MZ351174	X97304.1	100	100
	H2406	MZ326666	KY488557.1	83	76		H2393	MZ351175	X97304.1	100	100
	H2420	MZ326667	KY488557.1	98	71		H2421	MZ351176	KX950836.1	98	100
SCV	H2395	MZ326668	MH129615.1	94	100	ToRSV	H2392	MZ351177	KM083892.1	82	99
	H2399	MZ326669	MH129615.1	99	100						

Strawberry mottle virus (SMoV), beet pseudo yellows virus (BPYV), strawberry pallidosis-associated virus (SPaV), tomato ringspot virus (ToRSV), strawberry mild yellow edge virus (SMYEV), strawberry vein banding virus (SVBV), strawberry crinkle virus (SCV), strawberry polerovirus 1 (SPV-1), apple mosaic virus (ApMV), strawberry chlorotic fleck virus (SCFaV), strawberry crinivirus 4 (SCrV-4), strawberry crinivirus 3 (SCrV-3), Fragaria chiloensis latent virus (FClLV) and Fragaria chiloensis cryptic virus (FCCV). Identity for GenBank accession number (ID), coverage (Cov). Blue shading, proportional to the value, highlights divergent isolates.

**Table 3:** Newly designed assays for detection of strawberry-infecting viruses.

Virus	Oligo Name	Sequence (5′ to 3′)	5’ Reporter	Target Region	Length
Strawberry crinkle virus	SCV-F1	ATAGGGAGGAAAAACATTATCCG		RdRp	175 bp
	SCV-F2	ATAGGGAGGAAAAACATCATCCG			
	SCV-F3	ATAGGGAGGAAAAACATAATCCG			
	SCV-F4	ATAGGGAGGAAGAATATCATCCG			
	SCV-R1	CATTGGTGGCAGACCCATCA			
	SCV-R2	CATTGGTAGCAGATCCATCA			
	SCV-P1	TGTCACAAGATGCTGGAAG	FAM		
	SCV-P2	TGTCACAGGATGCTGGAA	FAM		
Strawberry mottle virus	SMoV-F1	GTAGTTTAGTGACAATCCAAGCGGA		3’ UTR	112 bp
	SMoV-F2	GTAGTTTAGCGACAATCCAAGCGGA			
	SMoV-R1	ATCCCACTTAGGGGCAAAGAA			
	SMoV-R2	ATCCCACTTAGGGGCAGAGGA			
	SMoV-P1	TAGGACACCGGCTCT	FAM		
Strawberry mild yellow edge virus	SMYEV-F1	AGGCTTAAAATGGGAGTTTCTTCTC		CP	83 bp
	SMYEV-F2	AGGCTTTAAATGGGAGTTTCTTCTC			
	SMYEV-F3	AGGCTTCAAATGGGAGTTTCTTCTC			
	SMYEV-R1	ACGGTTAAGTAGTACTATATTCACTTCATGG			
	SMYEV-R2	ACGGTTAAGTAGTACTACATTCACTTCATGG			
	SMYEV-P1	TTCTTCTACCCAGCTCC	FAM		
Strawberry pallidosis- associated virus	SPaV-F1	TTTGTATTCTGGTATGAATTTGGAAACT		Polyprotein 1b	123 bp
	SPaV-F2	TCTTTATTCTGGGATGAATTTAGAGACT			
	SPaV-R1	ACACCTTGAGATTTATCAAATTTGCT			
	SPaV-R2	ACACCTTGGGATTTATCGAATTTACT			
	SPaV-R3	ACACCTTGAGATTTATCGAACTTGCT			
	SPaV-P1	TTCCAATTCAAGAGTACAGGAC	FAM		
	SPaV-P2	TCCCAATTCAAGAGTATCGAAC	FAM		
Strawberry vein banding virus	SVBV-F1	TTTCTGTGACTATGAAACCAATCTTCT		CP	99 bp
	SVBV-F2	TTTCTGTGATTATGAAACCAATCTTCT			
	SVBV-R1	TCCATCCCGACAAAGGGTATT			
	SVBV-R2	TCCATTCCCACAAAGGGTATT			
	SVBV-R3	TCCATCCCAACAAAGGGTATT			
	SVBV-P1	CCTATAGAAGAATGGCCCAA	FAM		
	SVBV-P2	CCAATAGAAGAATGGCCCAA	FAM		
Strawberry polerovirus 1	SPV1-F1	GCCAGACGTCCCAGAGGTT		CP	61 bp
	SPV1-R1	GTCTTCCGGCATTCTGTTTCTT			
	SPV1-P1	AGAGGAGGAAAAGGAAA	FAM		
Beet pseudo yellows virus	BPYV-F1	AACGGTTGCAAGGTCAACATT		CP	62 bp
	BPYV-R1	GTTTCACGCTGTAGCCAATTTG			
	BPYV-R2	GTTTCACGCAGTAGCCAATTTG			
	BPYV-R3	GTTTCGCGCTGTAGCCAATTTG			
	BPYV-P1	CGGATGATGATTTGACG	FAM		
Tomato ringspot virus	ToRSV-F1	CCTGCAGAAGCAGATTGGC		CP	140 bp
	ToRSV-F2	CCTGCAGAAGCTGATTGGC			
	ToRSV-F3	CCTGCGGAAGCTGATTGGC			
	ToRSV-R1	GTTGGCCCGCGCCA			
	ToRSV-R2	GTAGGCCCGCGCCA			
	ToRSV-R3	GTTGGCCCACGCCA			
	ToRSV-P1	GGCGGTTCCTTTTATT	FAM		
	ToRSV-P2	CGCCGTTCCTTTTATT	FAM		
Apple mosaic virus	ApMV-F1	AAGCGAACCCGAATAAGGGT		CP	202 bp
	ApMV-F2	AGCGAACCCGAACAAGGG			
	ApMV-F3	GCGAACCCGAATAAGGGTAAA			
	ApMV-F4	GCGAACCCGAACAAGGGTA			
	ApMV-R1	CGGAAGACATCGGCAAAGTC			
	ApMV-R2	ACGAAAGACATCGGCAAAGTC			
	ApMV-P1	CTTGGGAGGTTAGAGGC	FAM		
	ApMV-P2	CTTGGGAGGTTAGTGTCC	FAM		
Strawberry chlorotic fleck virus	SCFaV-F1	TGAACTTGATCTGGGGCGTC		CP	196 bp
	SCFaV-R1	CCTCGAGCCCAGTGTCTCA			
	SCFaV-P1	AGGTTTGAAAAGAACCC	FAM		
Strawberry crinivirus 3	SCrV3-F1	CAACCACCGTCTACAAACATTACAA		Polyprotein 1a	126 bp
	SCrV3-R1	GGACCACGGTCGGAAGTTG			
	SCrV3-P1	TTTGTAGAACGCAGGCACAGT	FAM		
Strawberry crinivirus 4	SCrV4-F1	TTTGGGATTTCAGATTTGGGC		Polyprotein 1a	155 bp
	SCrV4-F2	ATAGGAATCTCGGATTTGGGC			
	SCrV4-R1	CGAGATCCAGATGAAGAAGTAACATAG			
	SCrV4-R2	CGAGATCCAGAGGAAGAAGTGAC			
	SCrV4-P1	TGTGAAGGAGAAGAATACCTA	FAM		
Fragaria chiloensis latent virus	FClLV-F1	CGTACTATCAATGAAACACTATCCAGAT		MP	189 bp
	FClLV-R1	TGGTTCAATCGGAACATTGTTC			
	FClLV-P1	CCATGTCTGATTTAGATAAA	FAM		
Fragaria chiloensis cryptic virus	FCCV-F1	CCTGACATAGCGTTCACACGA		RdRp	96 bp
	FCCV-R1	AAGTAGGACGTAATGAAACGCCTC			
	FCCV-P1	CAAATCAAGGTGAAGAGAA	FAM		

Forward primer (F), reverse primer (R), and TaqMan™ MGB probe (P). Coat protein (CP), movement protein (MP), RNA-dependent RNA polymerase (RdRp), and untranslated region (UTR). Default parameters for primer and probe Tm are 58–60 °C and 68–70 °C, respectively.

## Data Availability

The data presented in this study are openly available in GenBank, reference numbers MZ320192-MZ320216, MZ326662-MZ326687 and MZ351164-MZ351177.

## Supplementary Materials

The following are available online at https://www.mdpi.com/article/10.3390/v13081442/s1, Figure S1: Standard curves generated from new RT-qPCR assays for strawberry viruses, Table S1: Sequencing data generated from strawberry samples, Table S2: Detection of strawberry viruses in NCGR samples via RT-qPCR, Table S3: Detection of strawberry viruses in different positive controls stored at Foundation Plant Services.
