# Supplementary material for: Sequencing a Strawberry Germplasm Collection Reveals New Viral Genetic Diversity and the Basis for New RT-qPCR Assays
Source: Viruses. 2021 Jul 24;13(8):1442. doi: 10.3390/v13081442 (PMC8402890; doi:10.3390/v13081442)
Supplement: Supplementary file 1 [file viruses-13-01442-s001.zip › viruses-1302743-supplementary/Figure S1.pdf]

viruses

ToRSV

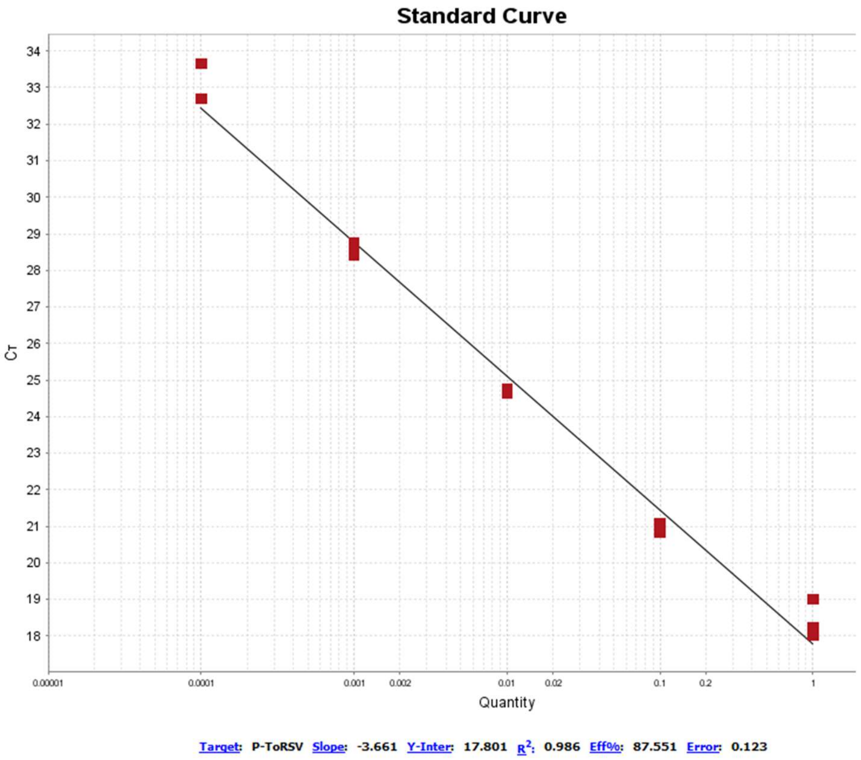

ApMV

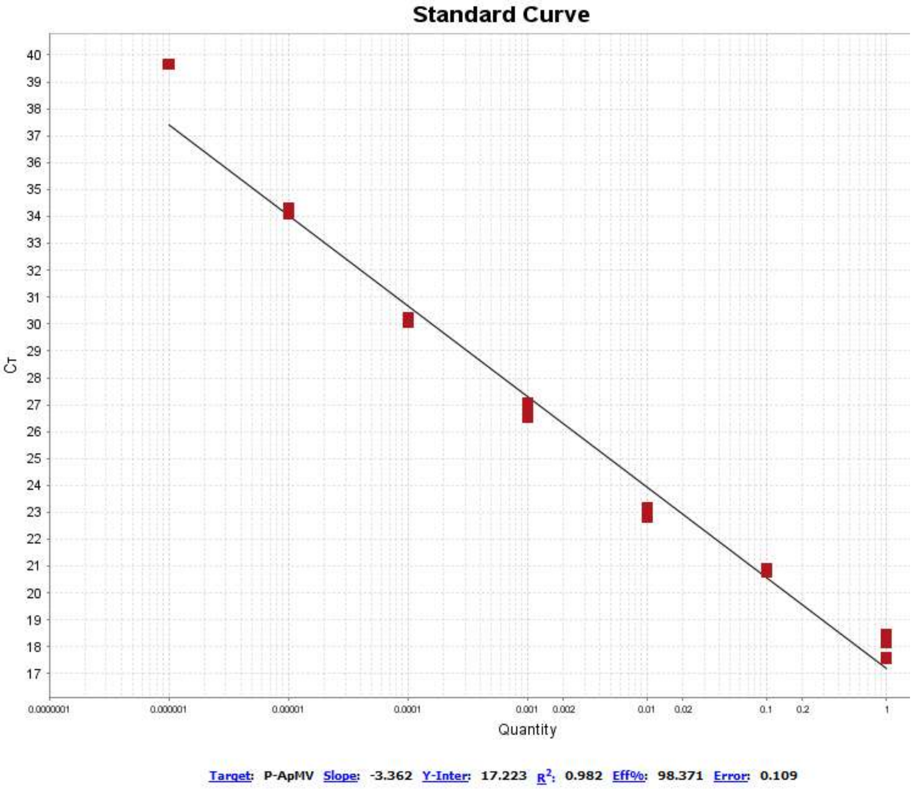

BPYV

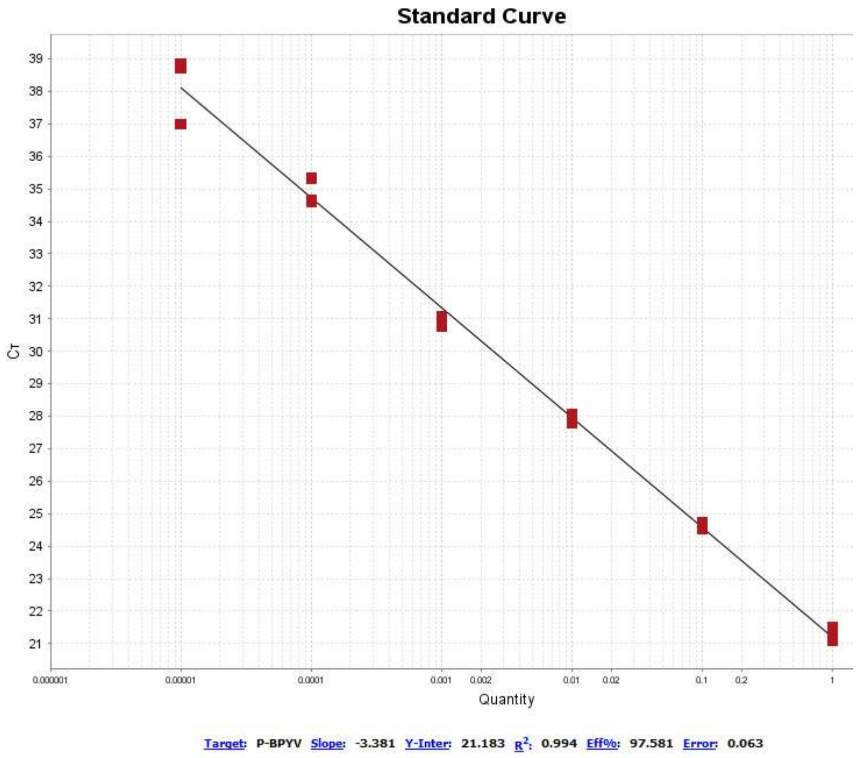
Standard Curve
CT
39 38 37 36 35 34 33 32 31 30 29 28 27 26 25 24 23 22 21
0.000001 0.00001 0.0001 0.001 0.002 0.01 0.02 0.1 0.2 1
Quantity
Target: P-BPYV Slope: -3.381 Y-Inter: 21.183 R2: 0.994 Eff%: 97.581 Error: 0.063

FCCV

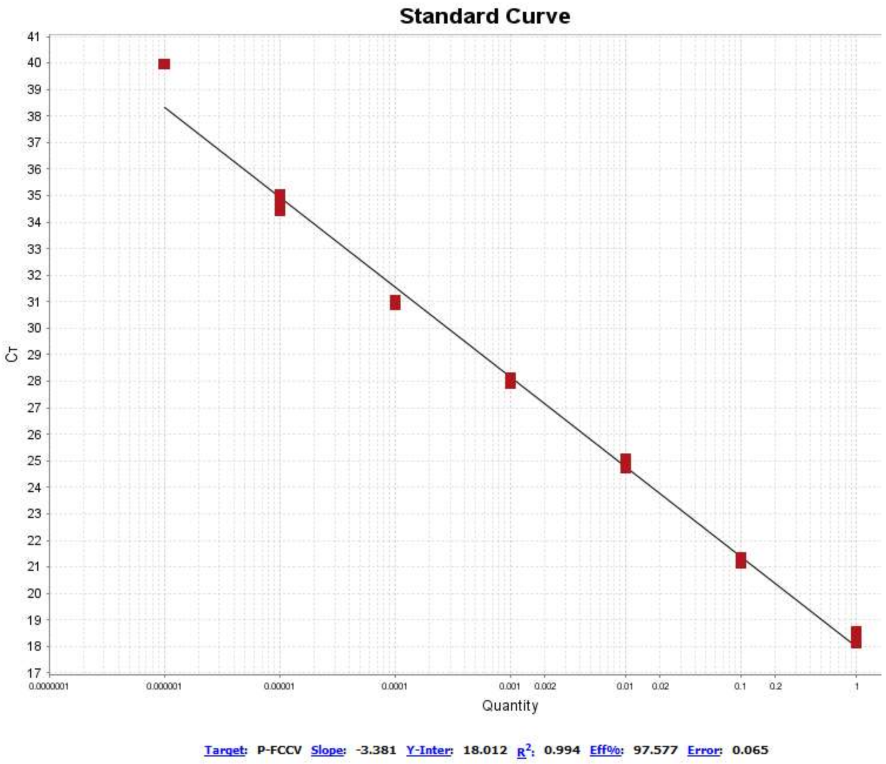
Standard Curve
CT
41 40 39 38 37 36 35 34 33 32 31 30 29 28 27 26 25 24 23 22 21 20 19 18 17
0.0000001 0.000001 0.00001 0.0001 0.001 0.002 0.01 0.02 0.1 0.2 1
Quantity
Target: P-FCCV Slope: -3.381 Y-Inter: 18.012 R2: 0.994 Eff%: 97.577 Error: 0.065

FClLC

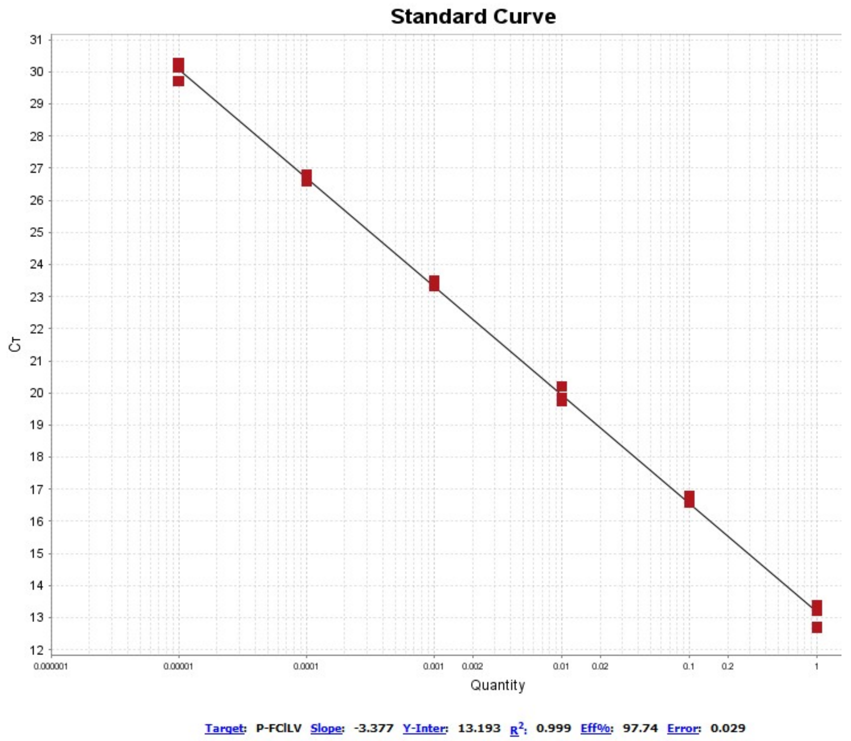
Standard Curve
CT
Quantity
Target: P-FClLV Slope: -3.377 Y-Inter: 13.193 R2: 0.999 Eff%: 97.74 Error: 0.029

SVBV

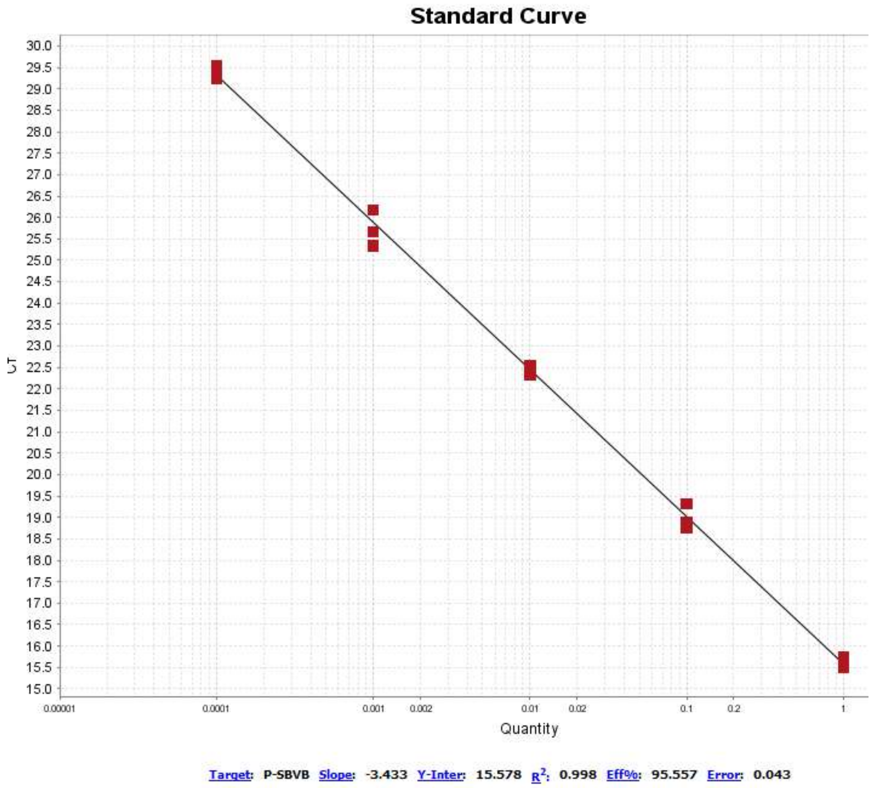
Standard Curve
CT
Quantity
Target: P-SBVB Slope: -3.433 Y-Inter: 15.578 R2: 0.998 Eff%: 95.557 Error: 0.043

SCFaV

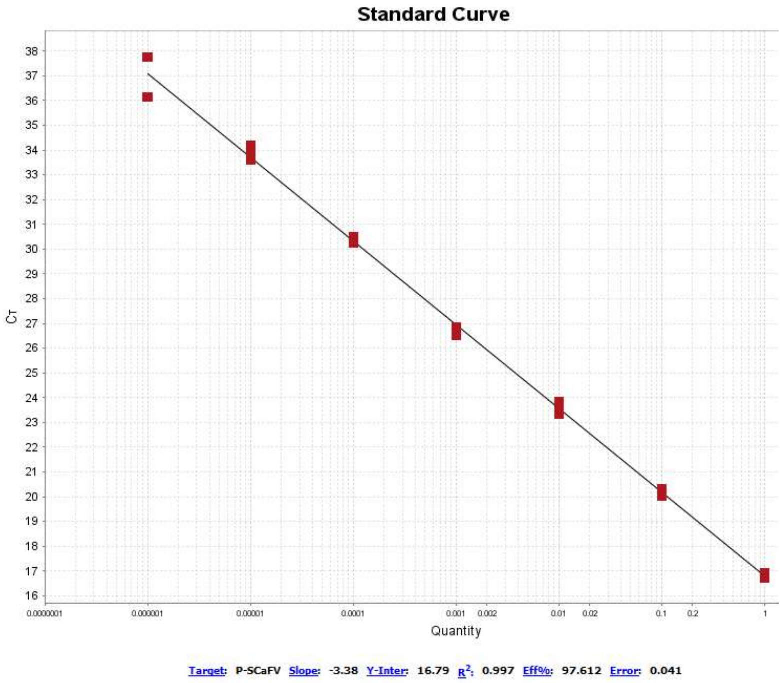
Standard Curve
CT
Quantity
Target: P-SCaFV Slope: -3.38 Y-Inter: 16.79 R2: 0.997 Eff%: 97.612 Error: 0.041

SCrV3

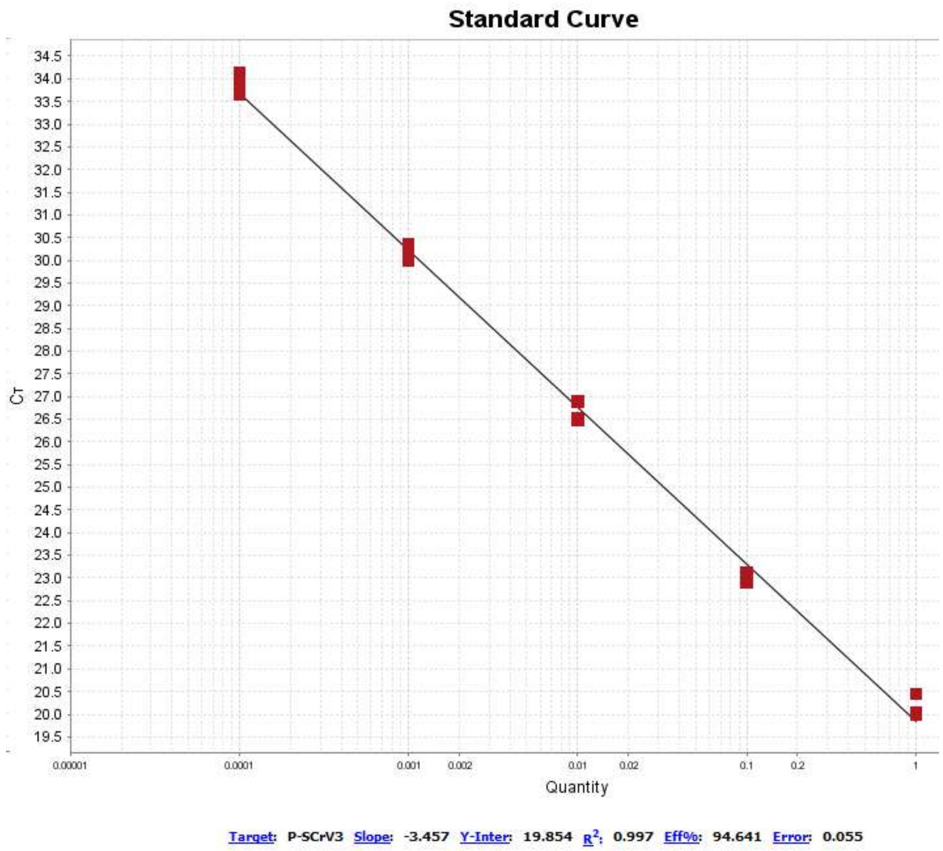
Standard Curve
CT
Quantity
Target: P-SCrV3 Slope: -3.457 Y-Inter: 19.854 R2: 0.997 Eff%: 94.641 Error: 0.055

SCrV4

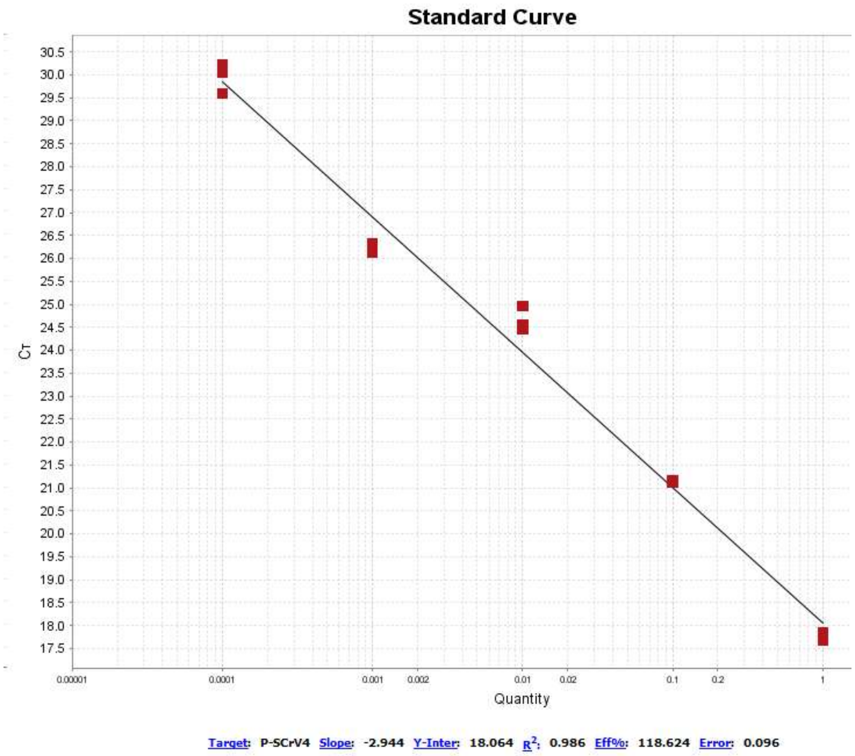
Standard Curve
CT
Quantity
0.00001
0.0001
0.001
0.002
0.01
0.02
0.1
0.2
1
Target: P-SCrV4 Slope: -2.944 Y-Inter: 18.064 R2: 0.986 Eff%: 118.624 Error: 0.096

SCV

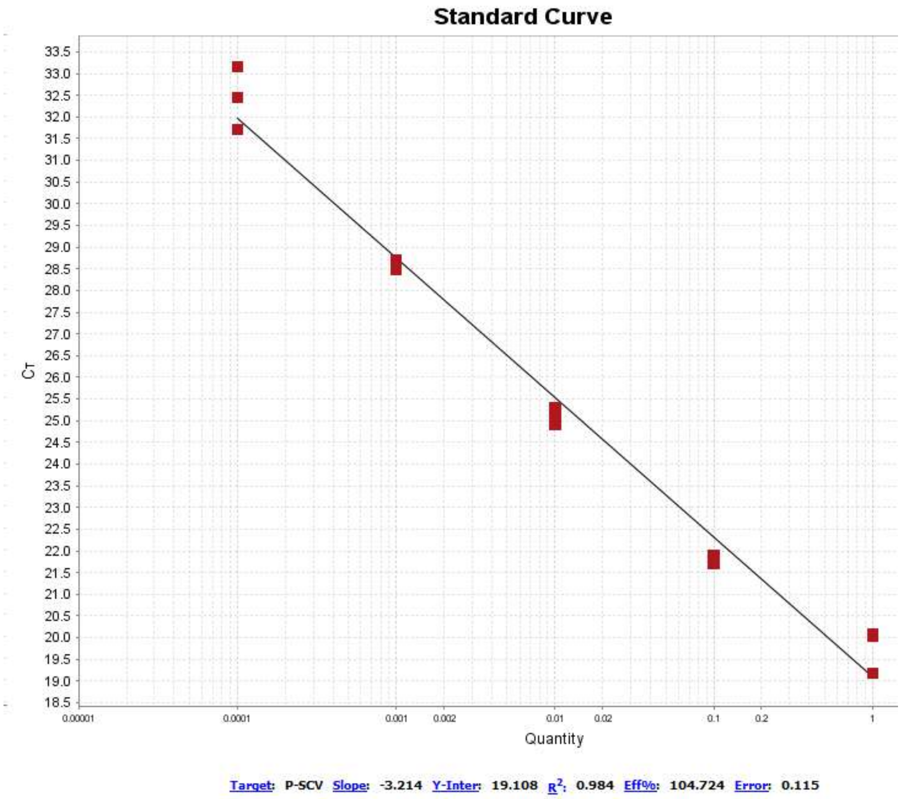
Standard Curve
CT
Quantity
0.00001
0.0001
0.001
0.002
0.01
0.02
0.1
0.2
1
Target: P-SCV Slope: -3.214 Y-Inter: 19.108 R2: 0.984 Eff%: 104.724 Error: 0.115

SMoV

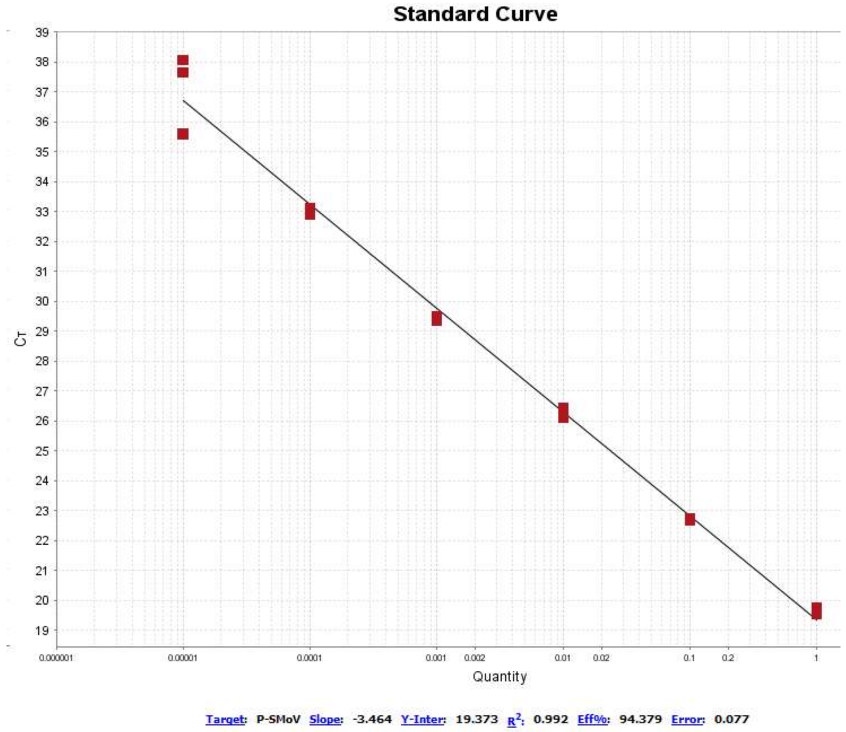
Standard Curve
CT
Quantity
Target: P-SMoV Slope: -3.464 Y-Inter: 19.373 R2: 0.992 Eff%: 94.379 Error: 0.077

SYMEV

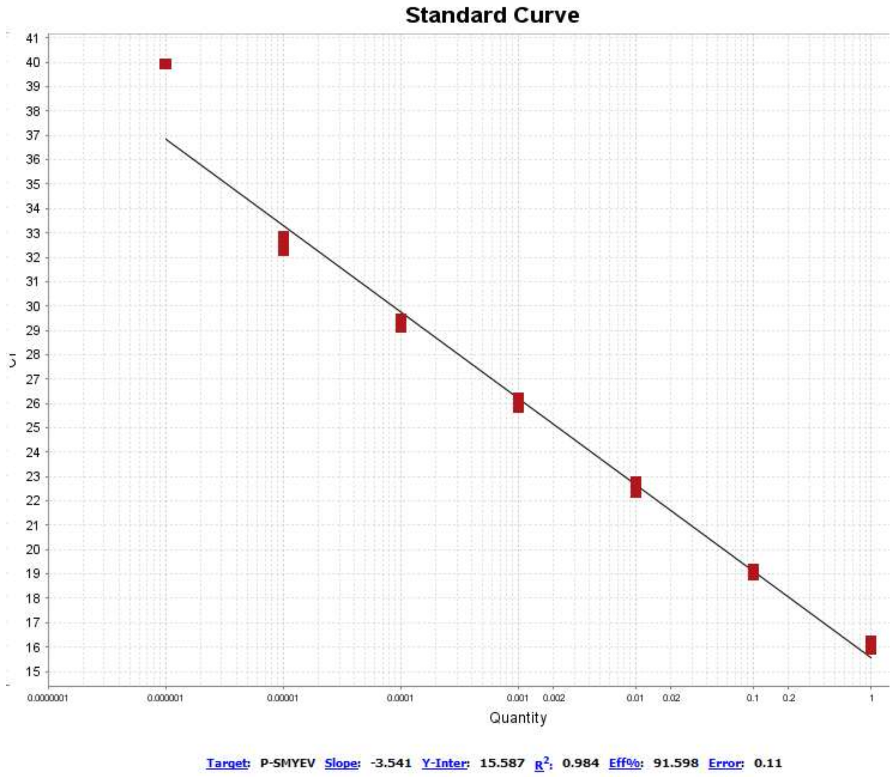
Standard Curve
Quantity
Target: P-SMYEV Slope: -3.541 Y-Inter: 15.587 R2: 0.984 Eff%: 91.598 Error: 0.11

SPV-1

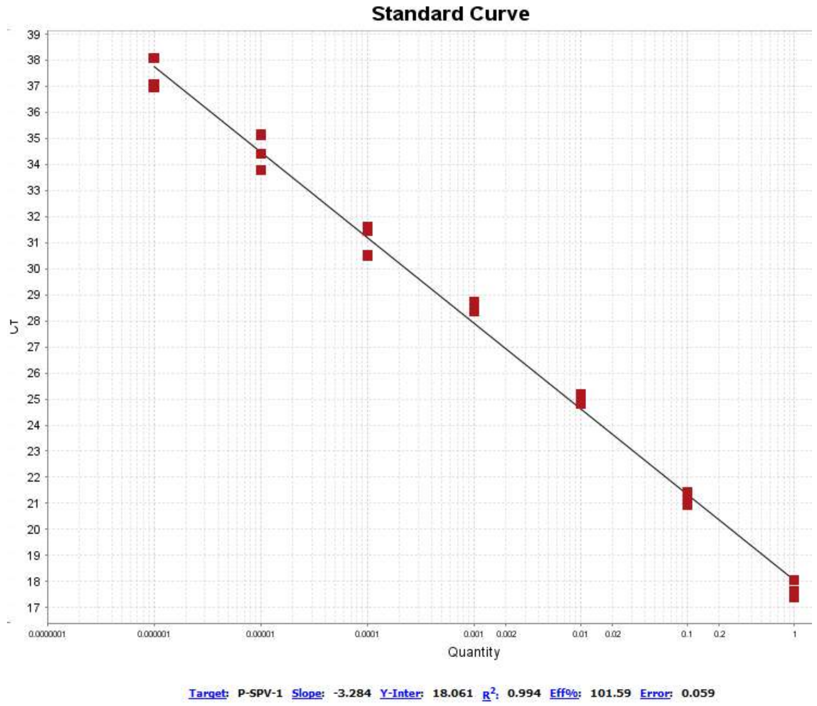

SPaV

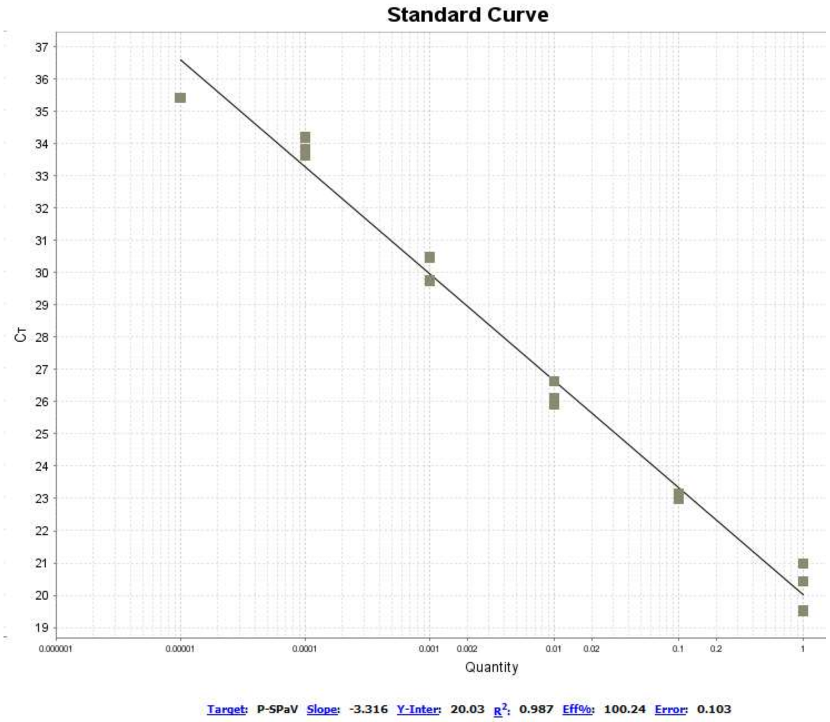

Figure S1. Standard curves generated from new RT-qPCR assays for strawberry viruses. Eff%, amplification efficiency in percentage; R2, coefficient of correlation.
